# Atypical Intestinal Metastases from Invasive Lobular Carcinoma of the Breast: Two Surgical Cases Mimicking Primary Gastrointestinal Tumors

**DOI:** 10.70352/scrj.cr.25-0554

**Published:** 2025-12-26

**Authors:** Yusuke Kitagawa, Hisanori Miki, Takumi Yamamoto, Yasuhiro Sakai, Jun Watanabe, Yosuke Fukunaga

**Affiliations:** 1 Department of Colorectal Surgery, Kansai Medical University Medical Center, Moriguchi, Osaka, Japan; 2 Department of Pathology, Kansai Medical University Medical Center, Moriguchi, Osaka, Japan; 3 Department of Colorectal Surgery, Kansai Medical University, Hirakata, Osaka, Japan

**Keywords:** invasive lobular carcinoma, gastrointestinal metastasis, immunohistochemistry, breast cancer, colonic stenosis

## Abstract

**INTRODUCTION:**

Gastrointestinal metastasis arising from invasive lobular carcinoma (ILC) of the breast is a rare but clinically significant manifestation. Unlike invasive ductal carcinoma, ILC has a higher propensity to metastasize into the gastrointestinal (GI) tract, often presenting with non-specific symptoms and mimicking primary GI tumors. Its accurate diagnosis requires a high index of suspicion and immunohistochemical (IHC) confirmation. Herein, we report 2 cases of intestinal metastases from ILC presenting with bowel stenosis.

**CASE PRESENTATION:**

Case 1 involved a 45-year-old woman with no known primary malignancy, who presented with duodenal and small bowel strictures. CT revealed narrowing of the small intestine, along with multiple sclerotic bone lesions. Surgical resection was performed, following which histopathological examination revealed ILC with a characteristic IHC profile (CK7^+^, CK20^–^, GATA3^+^, CDX2^–^, E-cadherin^–^). Retrospective breast imaging and biopsy confirmed a diagnosis of primary ILC. Case 2 involved a 57-year-old woman with a known history of luminal-type ILC. At 56 months postoperatively, bone metastasis was detected and PET-CT revealed uptake in the sigmoid colon. Furthermore, colonoscopy demonstrated stricture with no visible mucosal lesions. Surgical resection was subsequently performed, following which IHC confirmed metastatic ILC with a receptor status (ER^+^, PgR^+^, HER2^–^) matching that of the primary tumor.

**CONCLUSIONS:**

These cases demonstrate the diagnostic challenges posed by GI metastases arising from ILC, particularly due to submucosal infiltration and lack of endoscopic findings. IHC plays a critical role in differentiating these lesions from primary GI tumors. Although surgical resection may not prolong survival, it is valuable for symptomatic relief and for establishing a definitive diagnosis. Surgeons should be aware of the risk of ILC metastasizing into the GI tract. In patients presenting with atypical intestinal stenosis—particularly those with a history of ILC—metastasis should be considered. In such cases, IHC-guided diagnosis is essential.

## Abbreviations

CA
: carbohydrate antigen
CDX
: caudal-type homeobox
CEA
: carcinoembryonic antigen
CK
: cytokeratin
ER
: estrogen receptor
GATA
: GATA binding protein
GI
: gastrointestinal
HER
: human epidermal growth factor receptor
IDC
: invasive ductal carcinoma
IHC
: immunohistochemistry
ILC
: invasive lobular carcinoma
NCC-ST-439
: National Cancer Center sialylated tumor-related antigen

## INTRODUCTION

Breast cancer is the most frequently diagnosed malignancy and a leading cause of cancer-related deaths in women worldwide, accounting for approximately 11.7% of all new cancer cases and 6.9% of cancer-related deaths in 2020.^1)^ While the bone, liver, lung, and brain are the most common metastatic sites, GI involvement is rare. Clinical reports estimate an incidence of 0.2%–1.7% for the latter, whereas autopsy studies suggest rates as high as 16%.^2,3)^ However, the frequency of GI metastasis varies by histological subtype. ILC exhibits a significantly higher propensity for intestinal metastasis than IDC, with reported rates of 4.5% and 1.1%, respectively.^4–6)^

This distinct metastatic pattern of ILC is believed to be associated with the loss of expression of E-cadherin, a transmembrane protein crucial for cell–cell adhesion. Beyond its structural function, loss of E-cadherin expression has been implicated in tumor progression and metastatic dissemination.^7)^ Clinically, GI metastases from ILC are difficult to detect because of non-specific symptoms and lack of overt mucosal abnormalities in endoscopy.^6,8)^ These lesions often appear submucosal or stenotic, mimicking primary GI tumors or even benign diseases.

Given this diagnostic challenge, IHC is essential for distinguishing metastatic breast carcinoma from primary colorectal adenocarcinoma. Typical profiles of metastatic breast cancer include CK7^+^, CK20^–^, CDX2^–^, and GATA3^+^, with the loss of E-cadherin expression specifically supporting an ILC origin.^9,10)^ Moreover, the receptor status may not always be congruent between primary and metastatic sites, further complicating diagnosis and treatment planning.^11,12)^

Herein, we report cases of sigmoid colon metastasis arising from luminal-type ILC of the breast that occurred 67 months after surgery. These cases highlight the importance of maintaining a high index of suspicion in patients with ILC who develop intestinal symptoms and underscore the diagnostic and clinical value of IHC and imaging in managing this rare but clinically significant manifestation.

## CASE PRESENTATION

### Case 1

**Fig. 1:**
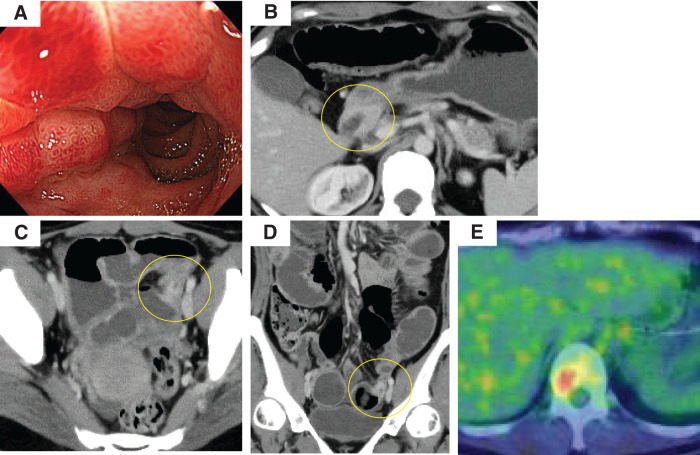
Imaging and endoscopic findings. (**A**) Upper gastrointestinal endoscopy showing mucosal erythema and edema with luminal narrowing in the duodenal bulb. (**B**) Contrast-enhanced CT demonstrating diffuse wall thickening with enhancement in the duodenum (yellow circle), corresponding to the endoscopic findings. (**C**, **D**) Axial (**C**) and coronal (**D**) contrast-enhanced CT images showing segmental wall thickening of the pelvic small intestine with luminal narrowing, leading to bowel obstruction (yellow circle). (**E**) PET-CT revealed abnormal uptake in the vertebral body, suggestive of bone metastasis.

**Fig. 2:**
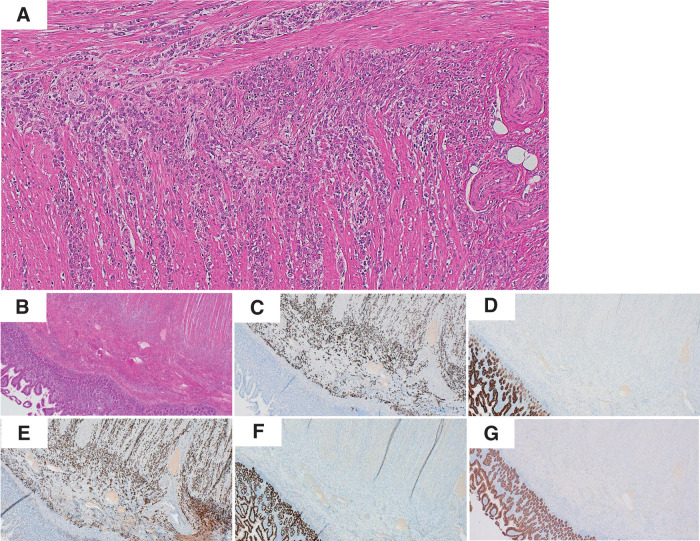
Histopathological and immunohistochemical findings of the small intestinal lesion. (**A**) HE staining (×100) revealed diffuse infiltration of small, uniform tumor cells arranged in linear cords within the submucosa and muscularis propria. (**B**) HE staining (×40) showed the lesion infiltrating the intestinal wall without mucosal surface involvement. (**C**) Immunohistochemistry for CK7 was strongly positive in tumor cells. (**D**) CK20 staining was negative. (**E**) GATA3 showed diffuse nuclear positivity, supporting a breast origin. (**F**) CDX2 staining was negative. (**G**) E-cadherin expression was lost in tumor cells, consistent with the characteristics of invasive lobular carcinoma of the breast. HE, hematoxylin and eosin

**Fig. 3:**
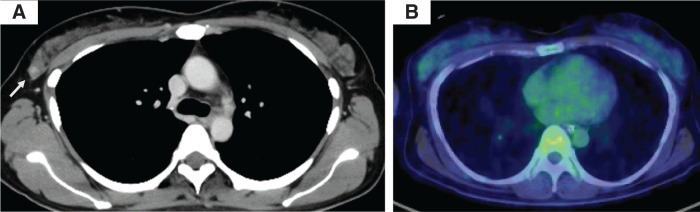
Retrospective evaluation of preoperative breast imaging. (**A**) Contrast-enhanced CT revealed a subtle, enhancing mass lesion in the outer quadrant of the right breast (arrow). (**B**) PET-CT showed faint FDG uptake in the corresponding area; however, this was not considered significant at the time of the preoperative assessment.

A 45-year-old female presented with epigastric pain that persisted for approximately 3 months. Upper gastrointestinal endoscopy performed at a referral hospital revealed edematous erythema of the duodenum, prompting further evaluation at our institution. Contrast-enhanced CT revealed a stricture in the duodenum, an additional narrowed area in the small intestine within the pelvic cavity, as well as multiple sclerotic bone lesions (**Fig. 1**). No primary tumor was identified at the time and tumor markers were observed to be within the normal ranges (CEA <1.0 ng/mL; CA19-9, 6.1 IU/mL; CA125, 68 U/mL). Consequently, the patient was referred to the surgical department for bowel obstruction relief. To this end, laparoscopic gastrojejunostomy and partial small bowel resection were performed. Subsequently, histological examination of the resected small intestine revealed findings suggestive of metastatic breast cancer of the ILC subtype. The tumor infiltration appeared to be in a cord-like pattern. Additionally, IHC revealed an expression profile of CK7^+^, CK20^–^, GATA3^+^, CDX2^–^, and E-cadherin^–^, confirming metastatic invasive lobular carcinoma (**Fig. 2**). Although no obvious breast mass was identified preoperatively, retrospective review of the contrast-enhanced CT images revealed a faintly enhanced lesion in the right breast (**Fig. 3**). Upon performing breast ultrasonography, the lesion appeared as an ill-defined, hypoechoic area. A core needle biopsy further confirmed the diagnosis of ILC. The final diagnosis was metastatic breast cancer involving the small intestine, duodenum, and bones. Currently, the patient is undergoing systemic chemotherapy for the same.

### Case 2

**Fig. 4:**
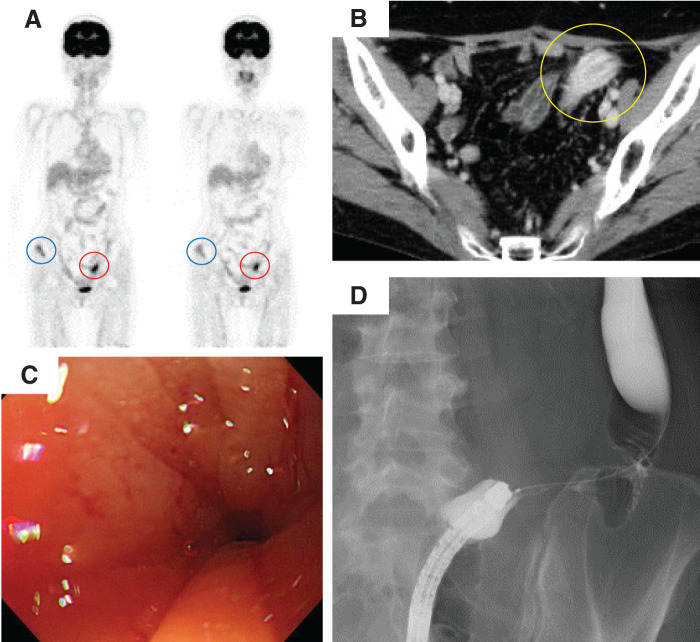
Imaging and endoscopic findings of sigmoid colon lesion. (**A**) PET-CT images showing FDG uptake in the right iliac bone (blue circles), as previously noted, as well as new accumulation in the sigmoid colon (red circles). (**B**) Contrast-enhanced CT revealed a mass lesion with enhancement in the sigmoid colon (yellow circle). (**C**) Colonoscopy demonstrated severe luminal narrowing in the sigmoid colon, preventing scope passage. No overt mucosal tumor was observed. (**D**) Contrast enema performed concurrently with colonoscopy revealed a tight stricture in the sigmoid colon, although a guidewire was able to pass through.

**Fig. 5:**
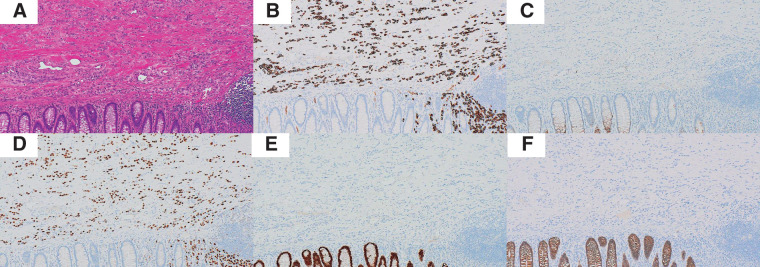
Histopathological and immunohistochemical findings of the sigmoid colon lesion. (**A**) HE staining showed diffuse infiltration of small, uniform tumor cells in the submucosa and muscularis propria (×10). (**B**) The tumor cells were diffusely positive for CK7. (**C**) CK20 was negative. (**D**) GATA3 was strongly positive. (**E**) CDX2 was negative. (**F**) E-Cadherin expression was lost, consistent with the characteristics of ILC. HE, hematoxylin and eosin; ILC, invasive lobular carcinoma

A 57-year-old female was diagnosed with luminal-type ILC of the right breast 67 months ago. She had undergone a mastectomy with sentinel lymph node dissection (pT3N0M0, Stage IIB). Thereafter, the patient received postoperative adjuvant therapy—including chemotherapy and endocrine therapy. After menopause, the hormonal agent was changed from tamoxifen to an aromatase inhibitor. At 56 months postoperatively, bone metastasis contiguous to the right iliac region was identified. The patient declined cytotoxic chemotherapy and was consequently administered fulvestrant. Eleven months later, PET-CT revealed increased uptake in the sigmoid colon, raising the suspicion of metastasis. Furthermore, colonoscopy revealed an endoscope-impassable stricture (**Fig. 4**) and the patient was referred for surgical intervention. She had no bowel obstruction, but required laxatives for defecation. The tumor marker levels were within the normal range (CEA, 4.8 ng/mL; CA19-9, 23.6 IU/mL; NCC-ST-439, 7.0 U/mL). Surgical resection of the sigmoid colon was performed following a multidisciplinary panel discussion. No peritoneal dissemination or ascites were observed. The postoperative course was uneventful, and the patient was discharged on POD 7. Subsequent histological examination revealed tumor infiltration in a cord-like pattern, extending from the mucosa to the subserosa. IHC analysis revealed the following expression profile: CK7^+^, CK20^–^, GATA3^+^, CDX2^–^, and E-cadherin^–^, confirming metastatic ILC (**Fig. 5**). Moreover, hormone receptor expression pattern (ER^+^, PgR^+^, and HER2^–^) was observed to be consistent with that of the primary breast tumor.

## DISCUSSION

We encountered 2 cases of intestinal metastases from ILC of the breast, both of which required surgical intervention owing to gastrointestinal stenosis. GI involvement in breast cancer is rare, occurring in only 0.2%–1.7% of clinical cases and up to 16% in autopsy series.^2,3)^ However, ILC demonstrates a unique predilection for metastasis to the GI tract, with reported intestinal metastasis rates of 4.5%, compared with only 1.1% in IDC.^4,5,10)^

Loss of E-cadherin expression is a hallmark of ILC and plays a central role in its distinctive growth pattern. E-Cadherin deficiency leads to discohesive, single-file infiltration of tumor cells and contributes to their ability to disseminate through fibrous and mucinous stromal components. In particular, ILC cells have been shown to preferentially spread along submucosal layers, where the loose connective tissue and mucin-rich extracellular matrix provide a permissive microenvironment for their infiltration.^3,13)^ This growth pattern often results in minimal mucosal involvement, making endoscopic diagnosis challenging. Consistent with these features, both our patients exhibited the characteristic immunophenotype of metastatic ILC: CK7^+^, CK20^–^, GATA3^+^, CDX2^–^, and E-cadherin^–^, confirming the lobular origin and metastatic nature of the tumor.^7)^ However, although this immunoprofile is highly suggestive of breast origin, it should not be considered pathognomonic. CK7/CK20 expression patterns are not exclusive to either breast or gastrointestinal origin, and false-negative results may arise when biopsy specimens are small or superficial—especially in the setting of submucosal infiltration.^14)^ GATA3, while commonly positive in breast carcinoma, has also been reported in non-breast tumors.^15)^ Therefore, immunohistochemical results must be interpreted in the context of clinical history, imaging findings, and morphological features, rather than relied upon solely. Another noteworthy issue is the discordance in the receptor status between primary and metastatic lesions. Treatment strategies for metastatic breast cancer are highly dependent on ER, PgR, and HER2 status, and reassessment of metastatic sites is crucial for optimal management. While both our patients demonstrated hormone receptor status consistent with that of the primary tumor (ER^+^, PgR^+^, and HER2^–^), previous studies have shown substantial discordance rates. Thus, it highlights the importance of reassessing receptor expression at metastatic sites when feasible.^11,12)^

The diagnosis of GI metastases arising from ILC can be challenging because the lesions frequently present as strictures without obvious mucosal abnormalities. In Case 1, no primary breast lesion was detected preoperatively and the diagnosis was established only after retrospective imaging and core needle biopsy following IHC analysis of the small intestine. In contrast, the patient in Case 2 had a known history of ILC, and PET-CT raised early suspicion of metastasis despite minimal symptoms. Colonoscopy revealed a fixed stricture with intact mucosa, consistent with prior reports that GI metastases from ILC often demonstrate submucosal or transmural infiltration rather than mucosal involvement, rendering endoscopic diagnosis difficult.^6,8)^

From a diagnostic perspective, colonic strictures can be broadly classified into 2 categories: those accompanied by mucosal abnormalities—such as primary colorectal cancer or inflammatory bowel disease—and those without apparent mucosal changes, including metastatic disease, ischemic, or post-radiation strictures.^16,17)^ In the latter group, standard mucosal biopsies frequently fail to obtain diagnostic tissue because tumor cells primarily infiltrate the submucosa or muscularis layer. Therefore, a comprehensive diagnostic approach incorporating clinical history taking, cross-sectional imaging, and, when necessary, surgical resection remains crucial for definitive diagnosis in these atypical presentations.

Surgical resection may offer both symptomatic relief and diagnostic confirmation in cases of GI metastasis, particularly when lesions are obstructive or biopsy results are inconclusive. In the context of oligometastatic breast cancer, local treatment—including surgical resection—has been associated with improved progression-free and overall survival in patients with extracranial oligometastases.^18)^ A multidisciplinary approach that integrates surgery, radiotherapy, and systemic therapy has also been suggested to be potentially beneficial in selected patients with limited metastatic burden.^19)^

Although GI metastasis from ILC has traditionally been considered a late manifestation with poor prognosis, recent findings indicate that such cases do not invariably represent terminal-stage disease. When systemic therapy remains effective and the disease is locally controllable, long-term survival may still be attainable,^20)^ supporting a more individualized treatment strategy for patients with ILC with GI involvement, including consideration of local therapies in the setting of oligometastases. These findings underscore the complex biological behavior of ILC and suggest that, even in cases with GI metastases, prognosis may be more favorable than previously assumed—particularly when aggressive but tailored interventions are employed. Further accumulation of high-quality case data is needed to refine treatment selection and better understand prognostic indicators.

## CONCLUSIONS

GI metastasis from ILC remains a major diagnostic and therapeutic challenge. Clinicians should maintain a high index of suspicion in patients with a history of ILC who present with non-specific GI symptoms. IHC remains indispensable for establishing a diagnosis, and surgical intervention may play an important role in symptom management as well as tissue diagnosis.
